# Docosahexaenoic acid inhibits the proliferation of Kras/TP53 double mutant pancreatic ductal adenocarcinoma cells through modulation of glutathione level and suppression of nucleotide synthesis

**DOI:** 10.1371/journal.pone.0241186

**Published:** 2020-11-02

**Authors:** Wei-Chia Hung, Der-Yen Lee, En-Pei Isabel Chiang, Jia-Ning Syu, Che-Yi Chao, Mei-Due Yang, Shu-Yao Tsai, Feng-Yao Tang

**Affiliations:** 1 Biomedical Science Laboratory, Department of Nutrition, China Medical University, Taichung, Taiwan, Republic of China; 2 Graduate Institute of Integrated Medicine, China Medical University, Taichung, Taiwan, Republic of China; 3 Department of Food Science and Biotechnology, National Chung Hsing University, Taichung, Taiwan, Republic of China; 4 Innovation and Development Center of Sustainable Agriculture (IDCSA), National Chung Hsing University, Taichung, Taiwan, Republic of China; 5 Department of Food Nutrition and Health Biotechnology, Asia University, Taichung, Taiwan, Republic of China; 6 Department of Medical Research, China Medical University Hospital, China Medical University, Taichung, Taiwan, Republic of China; 7 Department of Clinical Nutrition and Department of Surgery, China Medical University Hospital, Taichung, Taiwan, Republic of China; Centro Nacional de Investigaciones Oncologicas, SPAIN

## Abstract

The treatment of cancer cells obtained by blocking cellular metabolism has received a lot of attention recently. Previous studies have demonstrated that *Kras* mutation-mediated abnormal glucose metabolism would lead to an aberrant cell proliferation in human pancreatic ductal adenocarcinoma (PDAC) cells. Previous literature has suggested that consumption of fish oil is associated with lower risk of pancreatic cancer. In this study, we investigated the anti-cancer effects of docosahexaenoic acid (DHA) in human PDAC cells *in vitro* and *in vivo*. Omega-3 polyunsaturated fatty acids (PUFAs) such as DHA and eicosapentaenoic acid (EPA) significantly inhibited the proliferation of human PDAC cells. The actions of DHA were evaluated through an induction of cell cycle arrest at G1 phase and noticed a decreased expression of cyclin A, cyclin E and cyclin B proteins in HPAF-II cells. Moreover, it was found that co-treatment of DHA and gemcitabine (GEM) effectively induced oxidative stress and cell death in HPAF-II cells. Interestingly, DHA leads to an increased oxidative glutathione /reduced glutathione (GSSG/GSH) ratio and induced cell apoptosis in HPAF-II cells. The findings in the study showed that supplementation of GSH or N-Acetyl Cysteine (NAC) could reverse DHA-mediated cell death in HPAF-II cells. Additionally, DHA significantly increased cellular level of cysteine, cellular NADP/NADPH ratio and the expression of cystathionase (CTH) and SLCA11/xCT antiporter proteins in HPAF-II cells. The action of DHA was, in part, associated with the inactivation of STAT3 cascade in HPAF-II cells. Treatment with xCT inhibitors, such as erastin or sulfasalazine (SSZ), inhibited the cell survival ability in DHA-treated HPAF-II cells. DHA also inhibited nucleotide synthesis in HPAF-II cells. It was demonstrated in a mouse-xenograft model that consumption of fish oil significantly inhibited the growth of pancreatic adenocarcinoma and decreased cellular nucleotide level in tumor tissues. Furthermore, fish oil consumption induced an increment of GSSG/GSH ratio, an upregulation of xCT and CTH proteins in tumor tissues. In conclusion, DHA significantly inhibited survival of PDAC cells both *in vitro* and *in vivo* through its recently identified novel mode of action, including an increment in the ratio of GSSG/GSH and NADP/NADPH respectively, and promoting reduction in the levels of nucleotide synthesis.

## Introduction

Pancreatic cancer is one of leading causes of cancer mortality globally [1]. Around 85% of pancreatic cancer patients belong to the subtype of pancreatic ductal adenocarcinoma (PDAC) [2, 3]. Patients with PDAC have a 5-year survival rate of only 8% [3]. More than 90% of PDAC patients have mutationally activated *Kras* oncogene [4]. Most PDAC cells have extensively reprogrammed metabolism which is driven by *Kras* mutation [5]. *Kras* oncogene mutation also leads to aberrant nucleotide synthesis in PDAC patients [6]. PDAC cells are dependent on glucose and glutamine to maintain their metabolisms for proliferation and regulate anti-apoptotic escape [5, 7]. Previous studies have suggested that suppression of *Kras* oncogene activity leads to the death of PDAC cells [8].

It is important to note that about 70% of PDAC patients also have a mutation of *TP53*, *a* tumor suppressor gene [9]. Mutant p53 protein plays a role in modulating oncogenic function and induces alteration in cancer cell progression [10]. Previous evidence has also illustrated a significantly worse outcome among groups with *TP53* mutation in PDAC patients [11]. Conventional chemotherapeutic agents such as cisplatin and gemcitabine (GEM) have been widely used in the treatment of PDAC patients. Gemcitabine is an important component, commonly used in the clinical management of pancreatic cancer although severe side effects and acquired resistance are seen widespread in it [12]. Therefore, it has drawn a lot of attention from scientists who aim to discover novel chemopreventive and chemotherapeutic agents.

In most living organisms, intracellular redox homeostasis is mostly regulated by a balance between reduced glutathione (GSH) and oxidative glutathione (GSSG) [13, 14]. In order to maintain cellular redox balance, conversion of GSSG to GSH takes place at the expense of NADPH [15]. GSH, an antioxidant tripeptide, consists of glycine, glutamine and cysteine [15]. The transsulfuration pathway is involved in providing cysteine and contributes to the synthesis of GSH [16, 17]. In the transsulfuration pathway, cystathionine -β-synthase (CBS) and cystathionase (CTH) proteins play important roles in the conversion of cysteine [18]. Cysteine is used in synthesis of downstream product GSH through glutathione synthase (GSS) [18]. xCT (SLC7A11), a membrane transporter, plays an important role in cystine/glutamate transportation and in the regulation of cellular redox homeostasis [19]. The promoter region of *SLC7A11/ xCT* gene contains NRF2 binding sites in the antioxidant response element (ARE), which gets activated in response to increased intracellular oxidative stress [20]. A recent study has indicated that the *xCT* gene is probably modulated by the JAK/STAT3 signaling pathway [21] and the activation of this pathway would inhibit the expression of *SLC7A11/ xCT* gene [21]. A previous study also demonstrated that accumulated mutant-p53 protein suppressed the gene expression of *SLC7A11/xCT* [22]. Modulation of xCT transporter expression leads to an alteration of intracellular cysteine/glutamate levels [19]. A change of GSH/GSSG balance makes mutant p53 cancer cells more susceptible to oxidative stress [22].

Fish oil is abundant in omega-3 polyunsaturated fatty acids (PUFAs) including, eicosapentaenoic acid (EPA) and docosahexaenoic acid (DHA). A recent study indicated that omega-3 PUFAs especially, DHA could inhibit the activation of STAT3 signaling pathway and the proliferation of human PDAC cells *in vitro* [23, 24]. Previous studies have demonstrated that consumption of fish oil has shown an improved muscle mass, a positive chemotherapeutic response and decreased chemotherapy toxicity in PDAC patients [25]. Therefore, it is of interest to evaluate the possible mechanisms by which DHA could induce cell death such as, by modulation of intracellular glutathione level, regulation of STAT3/xCT signaling pathway and modification in cellular metabolism cascades. Hence, in this present study our aim is to demonstrate a novel anti-cancer mechanism of DHA, based on the mechanism of suppression of cell proliferation which is associated with modulation of GSSG/ GSH ratio and nucleotide synthesis in PDAC cells both *in vitro* and *in vivo*.

## Materials and methods

### Reagents and antibodies

Human PDAC HPAF-II (CRL-1997) and HPAC (CRL-2119) cells were authenticated and acquired from American Type Culture Collection (ATCC) (Manassas, VA). HPAF-II cells are characterized as pancreatic adenocarcinoma with *Kras*, *CDKN2A* and *TP53* gene mutation [26]. HPAC cells are characterized as pancreatic adenocarcinoma with *TP53* wild type and with *Kras* gene mutation. The following antibodies were obtained from Santa Cruz Biotech Inc. (Dallas, TX): anti-cyclin A, anti-cyclin B, anti-cyclin E, anti-phospho-EGFR (p-EGFR; Tyr1068), anti-phospho-c-Met (p-c-Met; Tyr1234/1235), anti-phospho-STAT3 (p-STAT3; Tyr705), anti-total-STAT3 (t- STAT3), anti-xCT, anti-CBS, anti-CTH, anti-GSS, anti-actin and anti-lamin A monoclonal antibodies. Lapatinib, PHA-665752, Ruxolitinib, ethanol, dimethyl sulfoxide (DMSO), 2’,7’-dichloro-dihydro-fluorescin diacetate (DCFDA), sulfasalazine (SSZ) and erastin were obtained from Sigma (St Louis, MO). DHA and EPA were purchased from Cayman Chemical Inc (Ann Arbor, MI). The nuclei and cytoplasm protein extract kits were purchased from Pierce Biotechnology Inc. (Lackford, IL). Propidium Iodide (PI) and anti-cyclin D1 antibody were acquired from BD Biosciences Inc. (Franklin Lakes, NJ). Minimum Essential Media (MEM), fetal bovine serum (FBS) and Annexin V-FITC Apoptosis Detection Kit were purchased from Invitrogen Inc. (Carlsbad, CA).

### Cell culture and treatment of omega-3 PUFAs

Human PDAC HPAF-II and HPAC cells were cultured and grown in MEM media supplemented with 10% FBS, 2 mM L-glutamine and 1.5 g/L sodium bicarbonate. PDAC HPAF-II and HPAC cells were incubated with various concentrations (0, 50, 100 and 150 μM) of the DHA or EPA for 24 or 48 h. For efficient treatment of PDAC cells, DHA or EPA was dissolved in ethanol and incorporated into FBS and mixed with the medium. In control groups, cells were incubated with an equivalent volume of solvent ethanol (final concentration: 0.05% v/v) as a carrier vehicle.

### Assessment of cell survival

Detection of cell survival analysis was performed using the trypan blue exclusion assay according to the previous protocol [27]. Human PDAC cells including HPAF-II cells or HPAC (2x 10^4^ cells / well) were seeded and grown in a 24-well plate until the treatment of DHA or EPA. These PDAC cells were cultured in media containing these omega-3 PUFAs (at concentrations of 0, 50, 100 and 150 μM) in the presence or absence of GEM. The cell survival assay was performed in a triplicate test. After 24 h and 48 h, cultured media were removed from each plate. Human PDAC cells were detached from each well by using trypsin/EDTA solution and then stained with trypan blue solution. The number of live (unstained) and dead (blue) cells were counted using a hemocytometer under a microscope. Cell viability represented the percentage of control subgroup.

#### Measurement of Reactive Oxygen Species (ROS)

The intracellular ROS level was determined by using the DCFDA assay according to previous established protocol [24]. Human HPAF-II cells were cultured in a 6-well plate and treated with DHA (0, 50, 100, 150μM) and GEM (10μM) respectively, for 24hrs. Once cells were washed with PBS, they were later incubated with DCFDA at a final concentration of 10 μM in MEM FBS free medium for 30 mins at 37°C. Cells included as positive control were cultured with H_2_O_2_ at a concentration of 1mM. After incubation, cells were collected and resuspended in 500μl phosphate buffer saline (PBS), then analyzed by using BD FACSCanto flow cytometry system (BD Biosciences Inc., Franklin Lakes, NJ). The DCFDA fluorescence intensity represented the intracellular ROS levels in HPAF-II cells.

### Analysis of cell cycle distribution

Human PDAC HPAF-II cells (1x10^6^ cells/well) were cultured in 3 cm culture plates. Cells were synchronized to the same cell cycle stage by culturing in MEM media with 0.5% FBS before initiating the experiment. To measure the effects of DHA on the cell cycle distribution, human PDAC HPAF-II cells were treated with DHA (0, 50 μM, 100 μM and 150 μM) for another 24 hours. Cells were detached with a trypsin/EDTA solution and later mixed with the binding buffer (1x10^5^ cells/mL). Suspended HPAF-II cells were then stained with PI in the dark and were analyzed using a FACSCanto flow cytometry system (BD Biosciences Inc., Franklin Lakes, NJ). The degree of PI-staining in human PDAC HPAF-II cells was measured with the help of an accessory software.

### Annexin-V-propidium iodide-binding assay

To determine whether DHA affected the apoptosis of PDAC HPAF-II cells, the apoptotic level of HPAF-II cells was detected by the Annexin V-FITC Apoptosis Detection Kit according to the manufacturer’s instruction. Briefly, after incubation with DHA at different concentrations (0, 50, 100 and 150 μM), 5X10^6^ isolated HPAF-II cells were resuspended in 500-μL-binding buffer. FITC-annexin V (5 μL) and propidium iodide (PI, 5 μL) working solution were added and then cells incubated at room temperature for 5 min. At the end of incubation period, cells were analyzed by flow cytometry, the annexin V-FITC binding was analyzed by FITC signal detector and PI staining by phycoerythrin emission signal detector.

### Protein extraction and Western blotting analysis

Protein extraction (cytoplasmic and nuclear proteins) of human HPAF-II cells were prepared using the Nuclear Protein Extract Reagent Kit containing inhibitors against protease and phosphatase. To remove the cell debris, cell lysates were centrifuged at 12,000 x g for 10 min. The upper phase of supernatants was kept as a cytoplasmic extract. The remaining precipitation was kept as a nuclear extract. Cellular proteins (60 μg) fractionated by 10% SDS-PAGE were transferred to a PVDF membrane and detected with anti-p-STAT3 monoclonal antibody. The remaining proteins in the cell lysates were measured using antibodies of anti- p-EGFR, anti-p-c-Met, anti-t-STAT3, anti-xCT, anti-CBS, anti-CTH, anti-GSS, anti-cyclin D1, anti-cyclin A, anti-cyclin B and anti-cyclin E. These blots were probed with internal control antibodies against actin or lamin A proteins.

### Analysis of thiol compounds and nucleotides

For the sample preparation of cellular metabolites, cells were seeded in a 6-well plate with at least 4–6 repeats in a density of 20x10^4^/well. For preparing metabolite samples from culture medium, 100 μL of culture media was extracted once it was treated well with 400 μL of methanol. The mixture was centrifuged at 14,000 rpm for 10 min and 400 μL of each sample was then transferred into a new 1.5 mL centrifuge tube. For preparing cellular metabolite samples, cells were extensively washed with 1XPBS (three times) so as to completely remove any residual PBS. This followed the addition of 300 μL of ddH_2_O into the plate in order to lyse cells and transfer the cell lysate into 1.5 mL centrifuge tubes. After centrifugation at 14,000 rpm for 10 minutes, 100 μL of cell lysate was mixed well with 400 μL of methanol. The mixture was again centrifuged at 14,000 rpm for 10 min and 400 μL of each sample was then transferred into a new 1.5 mL centrifuge tube. The remaining cell lysate was kept at a temperature of -80°C for further protein normalization. Extracted metabolite samples stored in 80% methanol were then vacuum-dried by a centrifugal concentrator and later kept frozen at a temperature of -80°C for further detection.

For the analysis of thiol compounds, metabolite samples were mixed with the reaction solution, containing 20 mM sodium carbonate (pH 9.5) and 0.2 mM ^13^C_6_-2-iodoacetaniline (^13^C_6_-2-IAN). The reaction mixture was incubated at 70°C for 2 hours and later terminated by the addition of 50 μL of 2% formic acid. Samples were centrifuged at 14,000 rpm for 10 min and the supernatants were subjected to analysis using the Vion IMS QTOF system.

For the analysis of nucleotides, cellular metabolite samples were mixed with the reaction solution, containing 30 μL of ddH_2_O, 5 μL of 0.3 M of aniline dissolved in HCl and 5 μ of 20 mg/mL N-(3-dimethylaminopropyl)- ethylcarbodiimide hydrochloride (EDC). The mixture was centrifuged at 14,000 rpm for 5 seconds, vortexed and incubated at room temperature for 2 hours. The reaction was terminated by adding 5 μL of 10% ammonium hydroxide followed by a 30 min incubation at room temperature. Then the samples were centrifuged at 14,000 rpm for 10 min and the supernatants were subjected to analysis using the Vion IMS QTOF system.

We briefly evaluated the process of using Liquid chromatography(LC)—electrospray ionization (ESI)- mass spectrometry(MS) analysis done in our study. The LC-ESI-MS system consisted of an ultra-performance liquid chromatography (UPLC) system (ACQUITY UPLC I-Class, Waters) and an ESI/APCI source of 4kDa quadrupole time-of-flight (TOF) mass spectrometer (Waters VION, Waters). The flow rate was set to 0.2 mL/min with a column temperature at 35°C. Separation was performed by using reversed-phase liquid chromatography (RPLC) technique on a BEH C18 column (2.1 x 100 mm, Walters) with 5 μL sample injection. The elution started initially from 99% mobile phase A (ultrapure water + 0.1% formic acid) and 1% mobile phase B (100% methanol + 0.1% formic acid), held at 1% B for 0.5 min, raised to 90% B in 5.5 min, held at 90% B for 1 min, and then lowered to 1% B in 1 min. The column was equilibrated by pumping 1% B for 4 min. LC-ESI-MS chromatogram was acquired by ESI+ mode which was kept under following conditions: capillary voltage kept at 2.5 kV, source temperature maintained at 100°C, desolvation temperature regulated at 250°C, cone gas maintained at 10 L/h, desolvation gas maintained at 600 L/h, and an acquisition by MSE mode with a range of m/z 100–1000 and a 0.5 s scan time. The acquired data was processed by UNIFI software (Waters) with an illustrated chromatogram and summarized in an integrated area of signals.

### Fish oil consumption in a mouse xenograft model of PDAC

A mouse xenograft model of PDAC was established through inoculation of HPAF-II cells. Briefly, confluent cultures of HPAF-II cells were lifted by treatment with trypsin/EDTA solution. Trypsinization of HPAF-II cell suspensions was terminated with FBS containing medium. Four-week old NOD SCID mice (body weight 18–24 g) were obtained from the National Animal Center in Taipei (Taiwan). The institutional Animal Care and Use Committee (IACUC) of China Medical University already approves this study (IACUC number: CMUIACUC-2018-052). This research including animal care and treatments is accorded with internationally accepted principles for animal use and care, as reviewed and approved by the IACUC Guidelines of China Medical University. These NOD SCID mice were raised in an institutional facility under specific pathogen-free (SPF) regulations. A Lab 5010 diet (from LabDiet Inc., St. Louis, MO, USA) was given to mice during the entire experimental period. All mice were anesthetized by inhalation of isofluorane during the experimental procedure according to animal protocol regulations. Viable HPAF-II cells (1 x 10^6^ cells/0.1 mL medium) were subcutaneously (s.c.) inoculated into the right flank of each NOD SCID mouse. After the transplantation of HPAF-II cells, these NOD SCID mice were randomly grouped into two subgroups (n = 6 in each subgroup). Fish oil (FO) were provided to those mice in the experimental subgroups by daily gavage at a total volume 0.15 mL. FO subgroups received a daily feeding of FO at dosages of 4% w/w, respectively. The tumor subgroup only received corn oil (4% w/w) in place of FO. An equation of 0·524 L1(L2)^2^ was used to measure tumor volume; where L1and L2 represented the long and short axis of the tumor tissues, respectively. To exclude dietary confounding factors, food intake and body weight (BW) of these mice were measured every week. We did not observe any significant differences in dietary intake or BW between the experimental (FO subgroup) and control (Tumor subgroup) subgroups. By the end of study, mice were euthanized with CO_2_ inhalation and sacrificed.

### Biostatistical analysis

A statistical analysis was used to determine the significant difference in the cell viability between control subgroup and experimental subgroups of PDAC cells using SYSTAT software. We used one-way ANOVA model to confirm a significant difference in cell viability which required an exclusion of null difference between the mean values originated from different subgroups at the *P* = 0.05 level. A Duncan's multiple range test was applied to evaluate differences among these subgroups.

## Results

### DHA inhibits cell survival through an induction of cell cycle arrest in human PDAC cells *in vitro*

**Fig 1:**
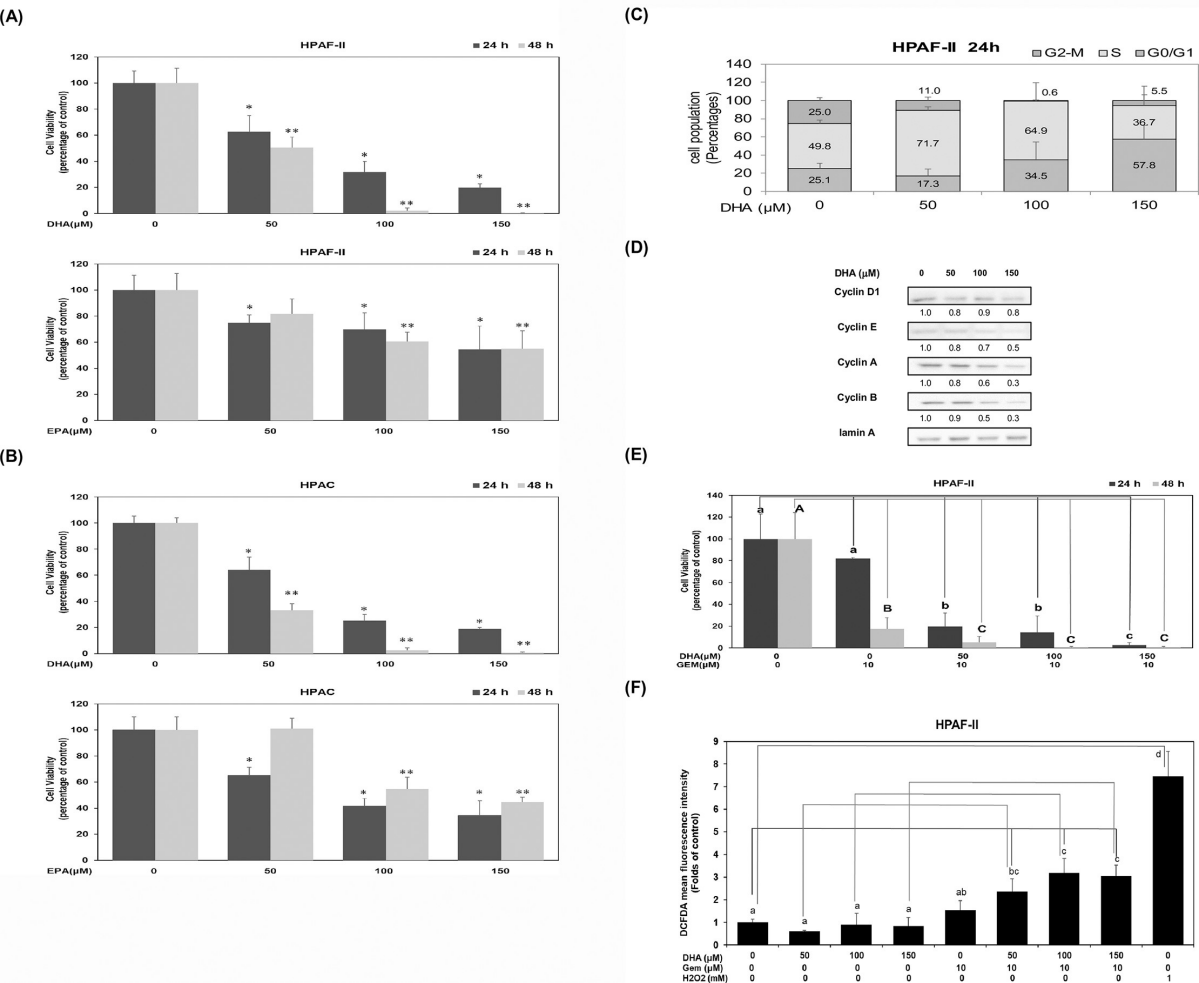
DHA inhibits cell survival through an induction of cell cycle arrest in human PDAC cells *in vitro*. Human PDAC cells such as HPAF-II (A) and HPAC (B) cells were cultured in MEM medium with DHA or EPA (at concentrations of 0, 50, 100 and 150 μM) for 24 hours (24 h) and 48 hours (48 h). Trypan blue exclusion assay was performed to measure cell viability as described in Materials and Methods. A single asterisk (*) represents a statistically significant difference in comparison with the untreated subgroup at 24 h, at *P* <0.05. A double asterisks (**) represents a statistically significant difference in comparison with the untreated subgroup at 48 h, at *P* <0.05. (C)To measure cell cycle distribution, human PDAC HPAF-II cells were cultured in the presence of DHA (0, 50, 100 and 150 μM) and cultured in 10% FBS MEM medium for 24 h. The measurement of the cell population at different cell cycle phases was performed using flow cytometry analysis, as described in Materials and Methods. (D) Human PDAC HPAF-II cells were treated with DHA (at concentrations of 0, 50, 100 and 150 μM) in 10% FBS MEM medium for 24 h. Western blot analysis of nuclear proteins was performed using monoclonal antibodies against cyclin D1, cyclin E, cyclin A, cyclin B and lamin A antibodies, as described under Materials and Methods. Band intensities represent the amounts of cyclin D1, cyclin E, cyclin A and cyclin B in the nuclei of human PDAC HPAF-II cells. (E) Human PDAC HPAF-II cells were cultured in MEM medium with DHA (at concentrations of 0, 50, 100 and 150 μM) in the presence or absence of 10 μM gemcitabine (GEM) for 24 h and 48 h. Trypan blue exclusion assay was performed to measure cell survival as described in Materials and Methods. Different lower-case letter represents a statistically significant difference within different subgroups at 24 h, at *P* <0.05 (indicated with blue line). Different upper-case letter represents a statistically significant difference within different subgroups at 48 h, at *P* <0.05 (indicated with red line). (F) Human PDAC HPAF-II cells were cultured in MEM medium with DHA (at concentrations of 0, 50, 100 and 150 μM) in the presence or absence of 10 μM gemcitabine (GEM) for 24 h. H_2_O_2_ (at a dosage of 1 mM) was used as a positive control. The intracellular ROS level was determined by using the DCFDA assay as described in Materials and Methods. Different letters represent a statistically significant difference among different subgroups at 24 h, at *P* <0.05. A blue line represents a significant difference of DHA_GEM groups in comparison with the untreated control group. Red lines represent a significant difference between DHA subgroups (at dosages of 50, 100 and 150 μM) in the absence or presence of GEM (10 μM). A green line represents a significant difference between the positive control group (H_2_O_2_ group) and untreated control group.

Human PDAC HPAF-II and HPAC cells were treated with DHA or EPA (at concentrations of 0, 50 μM,100 μM and 150 μM) for analysis of cell viability at different time points (24 hours and 48 hours). In comparison with the control subgroup, DHA at dosages of 50 μM, 100 μM and 150 μM, dose-dependently inhibited cell survival of HPAF-II cells up to 37.3%, 68.4% and 80.3%, respectively after 24 hours, and up to 49.5%, 97.9% and 99.5% after 48 hours, respectively (Fig 1A). It was detected that EPA at these three different concentrations inhibited cell survival of HPAF-II cells up to 25.2%, 30.2% and 45.7%, respectively after 24 hours and up to 18.3%, 39.4% and 45%, respectively at 48 hours (Fig 1A).

It was noticed that DHA at similar dosages (50 μM, 100 μM and 150 μM), dose-dependently suppressed cell survival of HPAC cells up to 36%, 74.7% and 81%, respectively after 24 hours and also reduced cell survival of HPAC cells up to 66.9%, 97.5% and 99.5%, respectively after 48 hours (Fig 1B). Additionally, it was seen that at these three different dosages EPA inhibited cell survival of HPAC cells up to 34.8%, 58.4% and 65.5%, respectively (24 hours). EPA at dosages of 100 μM and 150 μM inhibited cell survival of HPAC cells up to 45.3% and 55.4% (48 hours) (Fig 1B).

These results suggest that omega-3 PUFAs effectively inhibits cell survival in human PDAC HPAF-II and HPAC cells. DHA is more effective than EPA in regulating the suppression of cell survival in human PDAC cells. DHA significantly inhibited cell survival not only in *Kras* mutation/*TP53* wild type HPAC cells but also in *Kras* mutation/ *TP53* mutation HPAF-II cells. Therefore, we adopted HPAF-II, a double mutation of *Kras/TP53* PDAC cell line, as a major cell model to examine the inhibitory actions of DHA in this study.

To further demonstrate the inhibitory action of DHA, we examined the cell cycle distribution pattern in PDAC HPAF-II cells. As shown in Fig 1C, DHA treatment (at a concentration of 150 μM) induced cell cycle arrest at G1 phase up to 57.8% of total cells in comparison with 25.1% in the control subgroup. These changes in the outcome were mainly due to the modulation of cell cycle distribution at S and G2 phases. Hence, we investigated the effects of DHA on the expression of cell cycle regulatory proteins. Results showed that DHA mainly inhibited the expression of cyclin E, cyclin A and cyclin B proteins (Fig 1D). It was suggested that DHA effectively inhibited cell proliferation of human PDAC HPAF-II cells by blocking cell cycle transition from G1 to S phase.

We investigated the synergistic effects of DHA and GEM, a chemotherapeutic agent, to assess the level of suppression of cell viability in human PDAC HPAF-II cells. Results showed that DHA and GEM significantly inhibited cell survival in HPAF-II cells (Fig 1E). In comparison with the control subgroup, GEM alone (at a dosage of 10 μM) significantly inhibited the proliferation of HPAF-II cells up to 17.9% and 82.4% at 24 hours and 48 hours, respectively (Fig 1E). The present study showed that co-treatment of DHA (at dosages of 50 μM, 100 μM and 150 μM) and GEM (at dosage of 10 μM) enhanced the inhibition of cell survival of HPAF-II cells up to 80.4%, 85.7% and 97.5%, respectively after 24 hours and up to 94.8%, 99.4% and 99.4%, respectively after 48 hours (Fig 1E). These results suggest that DHA effectively enhanced the sensitivity of GEM and suppressed cell survival in human PDAC HPAF-II cells. In addition, we examined whether co-treatment of DHA and GEM would induce cell death by modulating oxidative stress level, with hydrogen peroxide (H_2_O_2_) being a positive control in our present study. The results showed that this co-treatment significantly induced an increment of oxidative stress level up to around 3.1 folds in human PDAC HPAF-II cells (*P*<0.05), as presented in Fig 1F.

### DHA inhibits cell survival through modulation of GSSG/GSH level in PDAC cells

**Fig 2:**
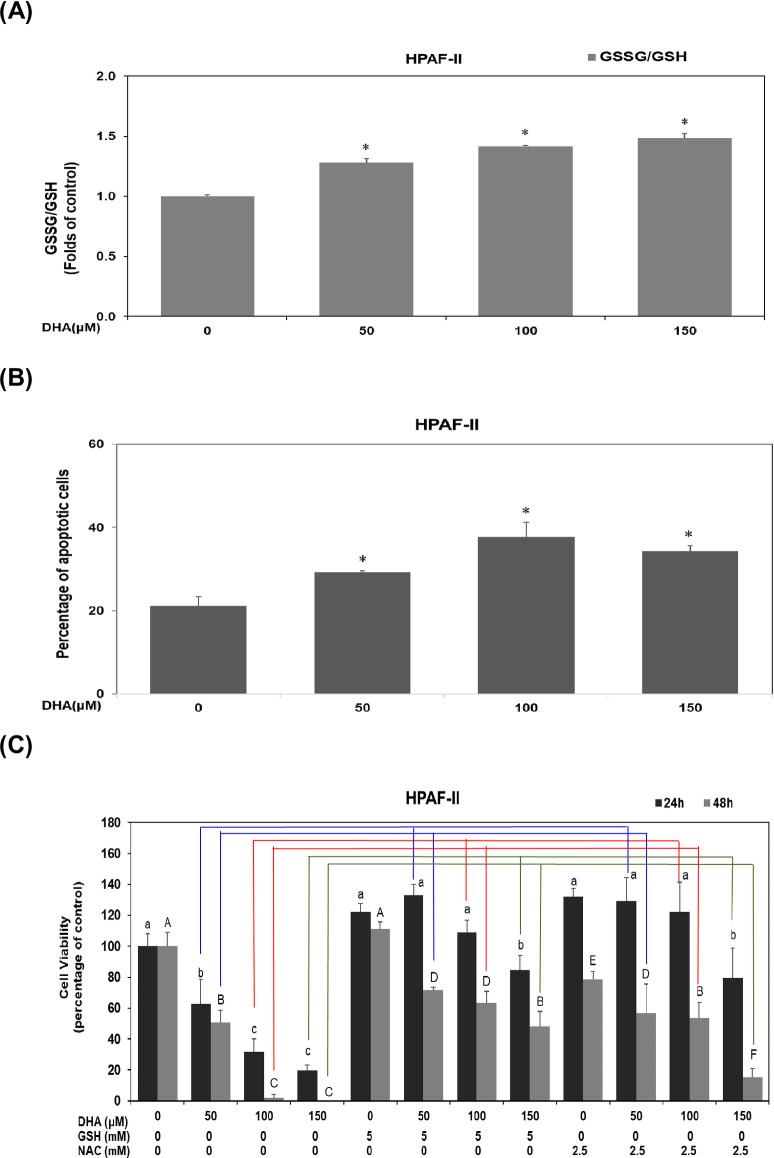
DHA inhibits cell survival through modulation of GSSG/GSH level in PDAC cells. (A) Human PDAC HPAF-II cells were cultured in MEM medium with DHA (at concentrations of 0, 50, 100 and 150 μM) for 24 h. Analysis of thiol compounds (GSSG and GSH) was performed as described in Materials and Methods. A single asterisk (*) represents a statistically significant difference in comparison with untreated subgroup of HPAF-II cells, at *P* <0.05. (B) The plots indicate the levels of apoptotic cell populations after treatment of DHA for 24 h in human PDAC HPAF-II cells. The quantitative result of the apoptotic cell populations was presented as apoptotic levels. Statistical significance is expressed as the mean ± SD (standard deviation) of three independent experiments. A single asterisk (*) represents a statistically significant difference in comparison with untreated subgroup of HPAF-II cells, at *P* <0.05. (C) Human PDAC HPAF-II cells were cultured in MEM medium with DHA (at concentrations of 0, 50, 100 and 150 μM) for 24 h and 48 h. The effect of GSH or NAC on DHA-treated HPAF-II cells were evaluated by using trypan blue exclusion assay described above. Different lower-case letter represents a statistically significant difference among different subgroups at 24 h, at *P* <0.05. Different upper-case letter represents a statistically significant difference among different subgroups at 48 h, at *P* <0.05. Blue lines represent a significant difference between DHA subgroups (at dosage of 50 μM) in the presence of GSH or NAC. Red lines represent a significant difference between DHA subgroups (at dosage of 100 μM) in the presence of GSH or NAC. Green lines represent a significant difference between DHA subgroups (at dosage of 150 μM) in the presence of GSH or NAC.

Previous studies have suggested a possible role of GSH status in cell cytotoxicity [28]. We investigated the underlying mechanism of action of DHA in HPAF-II cells. In this study, DHA at dosages of 50 μM, 100 μM and 150 μM, dose-dependently induced an increment of GSSG/GSH ratio up to 1.3, 1.4 and 1.5 folds, respectively, in human PDAC HPAF-II cells (Fig 2A). In comparison to the control group, treatment done by DHA at different concentrations (50 μM,100 μM and 150 μM) significantly enhanced apoptotic levels up to 29.2%, 37% and 34.3%, respectively (Fig 2B). To further verify these effects, we investigated the probable course of action by treating HPAF-II cells with 5 mM GSH or 2.5 mM N-Acetyl Cysteine (NAC). As shown in Fig 2C, it was revealed that supplementation of either GSH or NAC significantly reversed DHA-mediated cell death at 24 -hour and 48 -hour time points in human PDAC HPAF-II cells (*P*<0.05). These results suggested that DHA effectively inhibited cell survival, in part, through a rise in GSSG/GSH ratio in human PDAC HPAF-II cells.

### DHA modulates the cellular NADPH level and the expression of xCT antiporter, phosphorylated STAT3, CBS and CTH proteins in PDAC cells

**Fig 3:**
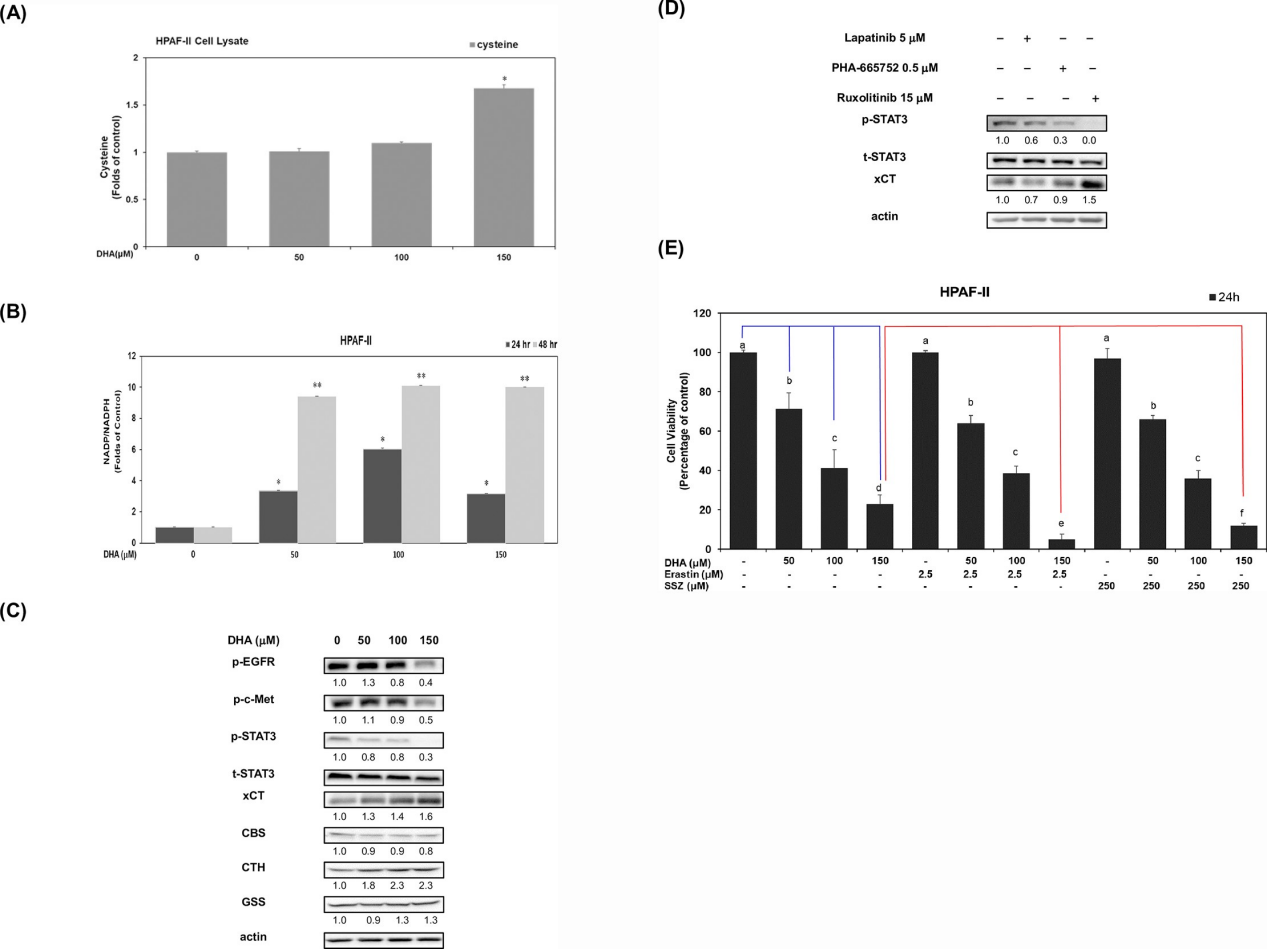
DHA modulates the cellular NADPH level and the expression of xCT antiporter, phosphorylated STAT3, CBS and CTH proteins in PDAC cells. Human HPAF-II cells treated with DHA (at concentrations of 0, 50, 100 and 150 μM) in 10% FBS MEM medium for 24 h. (A) Analysis of cellular cysteine level was performed as described in Materials and Methods. A single asterisk (*) represents a statistically significant in comparison with untreated subgroup of HPAF-II cells, at *P* <0.05. (B) Analysis of cellular NADP/NADPH level was performed as described in Materials and Methods. A single asterisk (*) represents a statistically significant difference in comparison with untreated subgroup of HPAF-II cells at 24 h, at *P* <0.05. A double asterisk (**) represents a statistically significant difference in comparison with untreated subgroup of HPAF-II cells at 48 h, at *P* <0.05. (C) Western blot analysis of cytoplasmic proteins (at 24 h time point) using monoclonal antibodies against p-EGFR (Tyr 1068), p-c-Met (Tyr1234/1235), p-STAT3 (Tyr705), t-STAT3, xCT, CBS, CTH, GSS and internal controls (actin). Band intensities represent the amounts of p-EGFR (Tyr 1068), p-c-Met (Tyr1234/1235), p-STAT3 (Tyr705), xCT, CBS, CTH and GSS in the cytoplasm of HPAF-II cells. (D) Human HPAF-II cells treated with Lapatinib (EGFR inhibitor, 5 μM), PHA-665752 (c-Met inhibitor, 0.5 μM) or Ruxolitinib (STAT3 inhibitor, 15 μM) for 24 h. Western blot analysis of cytoplasmic proteins (at 24 h time point) using monoclonal antibodies against p- p-STAT3 (Tyr705), t-STAT3, xCT and actin. Band intensities represent the amounts of p-STAT3 (Tyr705) and xCT in the cytoplasm of HPAF-II cells. (E) HPAF-II cells were pretreated with 2.5 μM erastin or 250 μM sulfasalazine (SSZ) for 24h and followed by treatment of DHA (0, 50, 100 and 150 μM) for 24 h. The effects of erastin or SSZ was measured by using trypan blue exclusion assay described above. Statistical significance is expressed as the mean ± SD (standard deviation) of two independent experiments. Different letters represent a statistically significant difference among different subgroups (at *P*<0.05). A blue line represents a significant difference between DHA subgroups (at dosages of 50, 100 and 150 μM) and the untreated control subgroup. A red line represents a significant difference between DHA subgroups (at dosage of 150 μM) in the presence of erastin or SSZ.

Previous literatures have indicated that intracellular cystine/cysteine is involved in the conversion of GSH [16]. Therefore, in present study we evaluated whether DHA modulates cellular cysteine levels and the expression of xCT protein in HPAF-II cells. The results showed that DHA significantly increased cellular cysteine level in HPAF-II cells (*P*<0.05) (Fig 3A). Previous studies demonstrated that NADPH is involved in the conversion of GSSG to GSH [29]. Based on our earlier findings, it is plausible that DHA modulates cellular NADPH level and blocks the conversion of GSSG to GSH [29]. To verify this hypothesis, we examined whether DHA treatment modulated cellular NADP and NADPH levels in HPAF-II cells. Results showed that DHA significantly induced an increment of NADP/NADPH ratio in HPAF-II cells (Fig 3B). These findings suggested that DHA effectively blocked the conversion of GSSG to GSH by increasing NADP levels in HPAF-II cells.

A previous study has indicated that the xCT antiporter protein is responsible for the transportation of cellular cysteine/glutamate [19]. Hence, we investigated whether DHA could modulate the expression of xCT antiporter protein and enzymes involved in the transsulfuration pathway. As shown in Fig 3C, DHA induced the expression of xCT protein in human PDAC HPAF-II cells. However, DHA slightly suppressed the expression of CBS protein. Interestingly, it was seen that DHA effectively induced an increased expression of CTH protein in human PDAC HPAF-II cells (Fig 3C). Our results also showed that DHA successfully inhibited the phosphorylation of EGFR, c-Met and STAT3 proteins in HPAF-II cells (Fig 3C). The mechanism of action was examined at molecular level by studying the treatment response with specific inhibitors against EGFR, c-MET or JAK/STAT3 signaling molecules. It was clearly seen that treatment with STAT3 inhibitor (Ruxolitinib) significantly induced the expression of xCT protein (Fig 3D). These results suggested that DHA augments the expression of xCT antiporter protein mainly by decreasing expression of phosphorylated STAT-3 protein. Interestingly, it was seen that pretreatment of PDAC cells with xCT inhibitors such as erastin or sulfasalazine (SSZ) effectively inhibited cell survival in DHA (150 uM) -treated HPAF-II cells (Fig 3E). The data suggested that DHA modulates cellular GSSG/GSH level which is associated with an increment of NADP/NADPH ratio in human PDAC HPAF-II cells. DHA slightly inhibited the expression of CBS protein in human PDAC HPAF-II cells. However, DHA increased cellular level of cysteine and the expression of xCT and CTH proteins. There is a possibility that DHA induces a feedback control which leads to an upregulation of xCT and CTH proteins along with an increase in intracellular cysteine level caused due to the lack of GSH in HPAF-II cells. The molecular mechanism of action behind this entire process, involved a reduced phosphorylation of STAT3 protein in HPAF-II cells.

### DHA suppresses the nucleotide synthesis in PDAC cells

**Fig 4:**
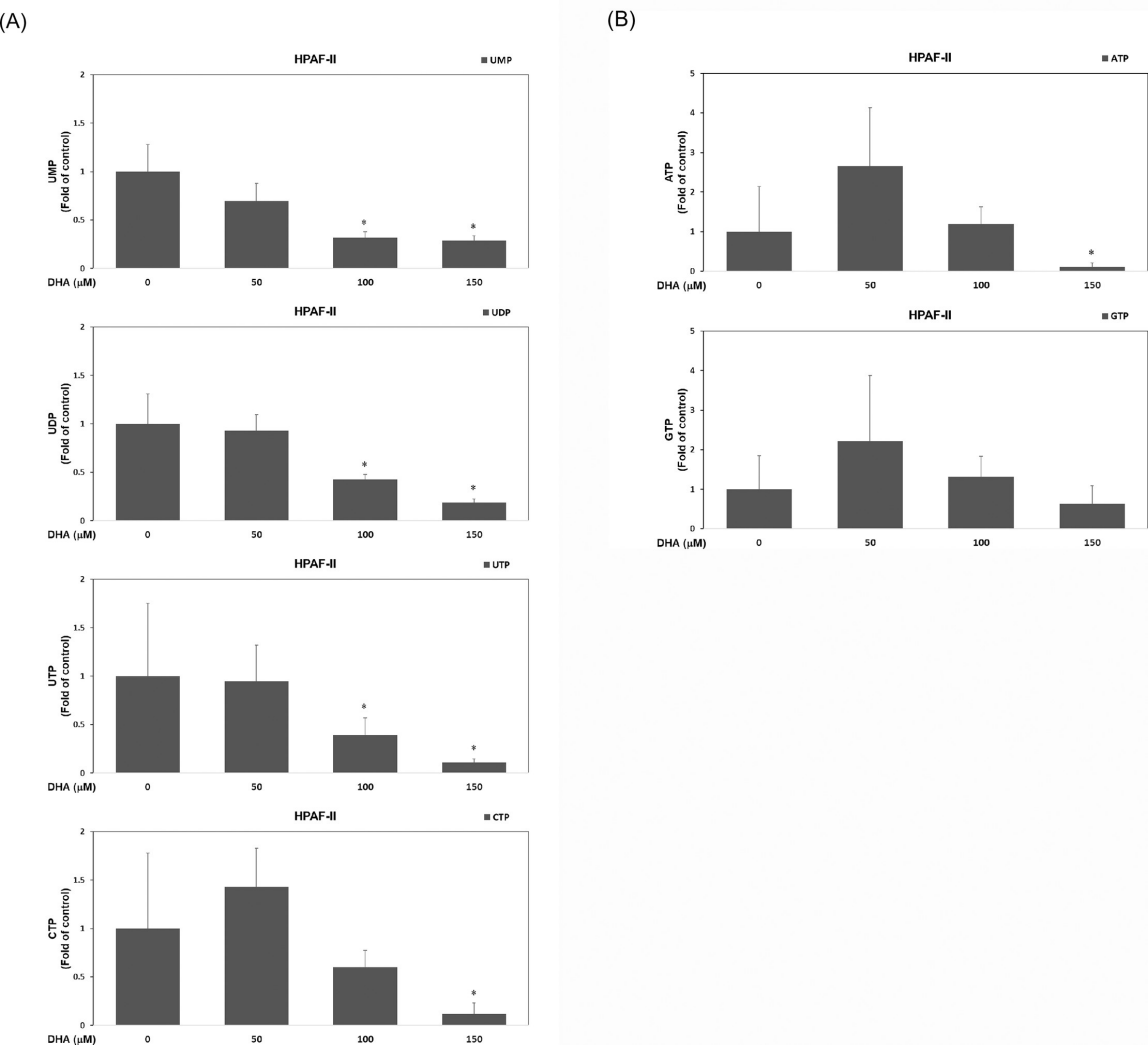
DHA suppresses the nucleotide synthesis in PDAC cells. (A) Human PDAC HPAF-II cells were treated with DHA (at concentrations of 0, 50, 100 and 150 μM) in 10% FBS MEM medium for 24 h. Analysis of cellular UMP, UDP, UTP and CTP levels was performed as described in Materials and Methods. A single asterisk (*) represents a statistically significant difference in comparison with untreated subgroup of HPAF-II cells, at *P* <0.05. (B) Analysis of cellular ATP and GTP levels was performed as described in Materials and Methods. A single asterisk (*) represents a statistically significant difference in comparison with untreated subgroup of HPAF-II cells, at *P* <0.05.

In our present study, the results clearly demonstrated that DHA significantly hampers cell cycle progression through a cell cycle arrest taking place during G1/S phase transition. Based on the assumption that DHA may suppress the synthesis of nucleotides in human PDAC HPAF-II cells, we studied whether it could modulate the synthesis of nucleotides in HPAF-II cells. The results in the study showed that DHA significantly reduced cellular levels of uridine monophosphate (UMP), uridine diphosphate (UDP), uridine triphosphate (UTP) and cytidine triphosphate (CTP) (Fig 4A) and also effectively suppressed cellular levels of adenosine triphosphate (ATP) (Fig 4B). The outcome has suggested that DHA might inhibit cell proliferation through suppression of nucleotide synthesis in HPAF-II cells. The result of the present study highlights that DHA inhibits cell proliferation by blocking nucleotide synthesis process.

### Consumption of fish oil inhibits the growth of pancreatic adenocarcinoma by suppressing nucleotide synthesis in a mouse xenograft model

**Fig 5:**
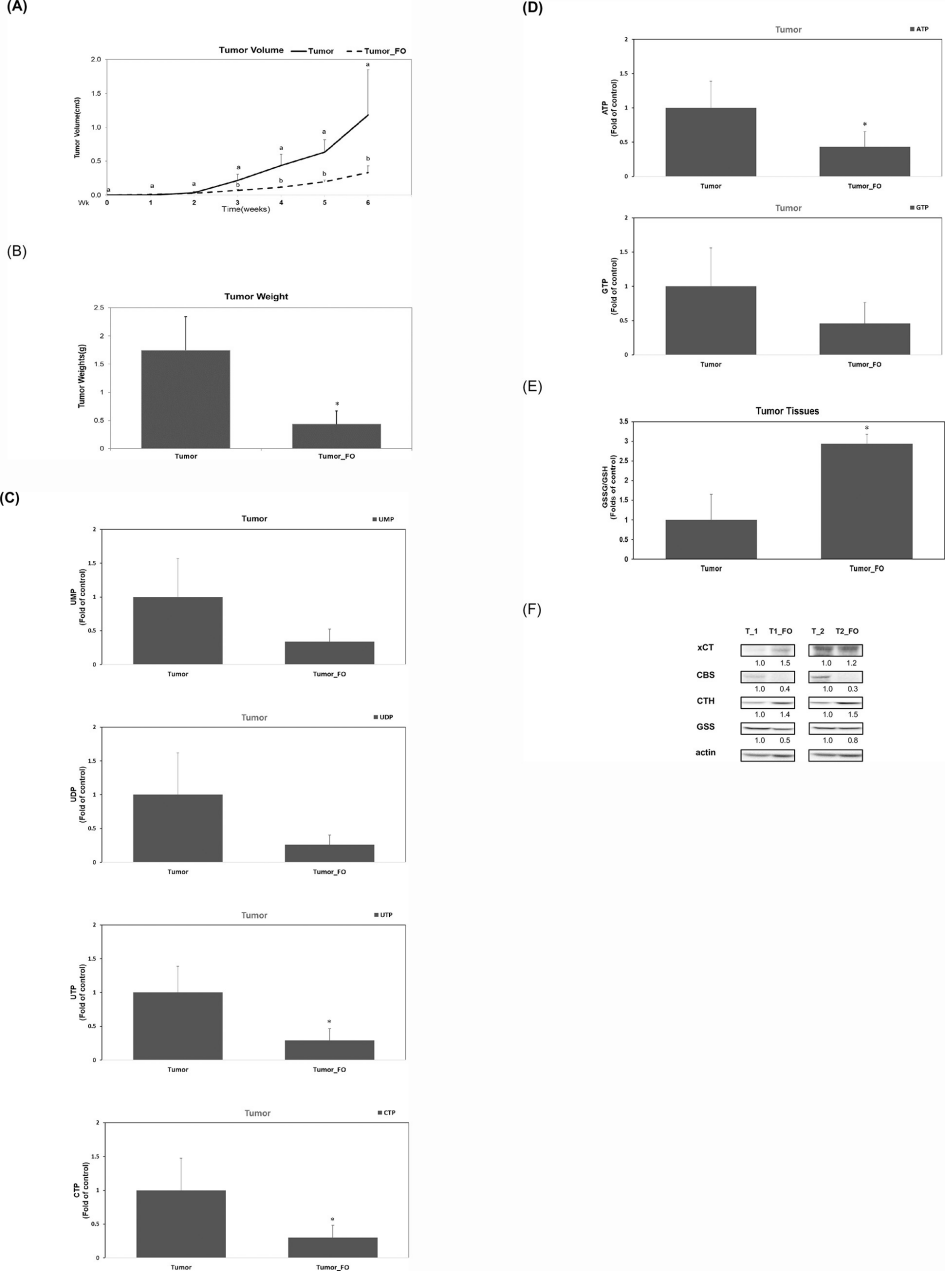
Consumption of fish oil inhibits the growth of pancreatic adenocarcinoma by suppressing nucleotide synthesis in a mouse xenograft model. Xenograft NOD SCID mice (n = 6 for each subgroup) were divided into two subgroups (the tumor subgroup and tumor_FO subgroup). The tumor_FO subgroup was given FO (at a dosage of 4% w/w diet per day) for 6 weeks. Data represent the change in the tumor volume (A) and tumor weight (B) between the tumor subgroup (Tumor; the control subgroup) and tumor with FO at dosage of 4% w/w diet per day (Tumor_FO; the experimental subgroup). (A) For the analysis of tumor volume, different letters represent a statistically significant difference in comparison to the control subgroup at the same time points *(P<0*.*05)*. (B) For the analysis of tumor weight, a single asterisk (*) indicates a significant difference in comparison to the control subgroup *(P<0*.*05)*. (C) Analysis of UMP, UDP, UTP and CTP levels in tumor tissues was performed as described in Materials and Methods. A single asterisk represents a statistically significant difference in comparison to the control subgroup, at *P* <0.05. (D) Analysis of ATP and GTP levels in tumor tissues was performed as described in Materials and Methods. A single asterisk represents a statistically significant difference in comparison to the control subgroup, at *P* <0.05. (E) Analysis of thiol compounds (GSSG and GSH) was performed as described in Materials and Methods. A single asterisk represents a statistically significant difference of GSSG/GSH ratio in tumor tissues in tumor_FO subgroup in comparison to the control subgroup, at *P* <0.05. (F) Western blot analysis of proteins in different sets of tumor tissues using monoclonal antibodies against xCT, CBS, CTH, GSS and internal controls (actin). Band intensities represent the amounts of xCT, CBS, CTH and GSS proteins.

To further verify these *in vitro* findings, we conducted an animal study to assess whether consumption of fish oil (FO) causes an inhibition of tumor growth. The study performed in a mouse xenograft model showed that FO consumption significantly inhibited tumor growth (Fig 5A) and tumor weight (Fig 5B). Data extracted from cell culture studies showed that DHA successfully inhibited nucleotide synthesis in human PDAC HPAF-II cells. Role of FO consumption in modulating nucleotide levels in tumor tissues was also assessed. The results demonstrated that FO consumption significantly reduced levels of UTP and CTP in tumor tissues *in vivo* (*P<0*.*05*), as shown in Fig 5C and also significantly inhibited ATP levels in tumor tissues (*P<0*.*05*) (Fig 5D). When compared with the untreated tumor group (control group), the group with FO consumption (experimental group) also showed higher GSSG/GSH ratio in tumor tissue (Fig 5E). This outcome is also justified as consumption of FO induces an upregulation of xCT and CTH proteins and reduces the expression of CBS proteins in tumor tissues (Fig 5F). The results verified that FO consumption inhibited tumor growth by modulating GSSG/GSH level, suppressing CBS protein expression, nucleotide synthesis and reducing energy source (ATP) in a HPAF-II cell- implanted mouse xenograft model.

## Discussion

*Kras* and *TP53* gene double mutation is a major cause of cancer progression in PDAC [9]. Mutation of *Kras* oncogene, induces metabolism addiction and increases nutrients uptake [5]. In addition, *TP53* mutation is widely associated with aggressive tumor phenotype expression and a poor survival rate in PDAC patients caused mainly due to accumulation of mutant-p53 protein or loss of transcriptional activity [30]. Therefore, effective therapeutic agents to target mutant-*TP53* cancer cells are in great demand.

Numerous epidemiological studies have indicated that consumption of omega-3 PUFA fish oil is significantly correlated with a lower risk of pancreatic cancer [31]. DHA and EPA, the major omega-3 PUFAs present in fish oil, have been suggested to be potential agents used in the treatment of inflammation and in prevention of various types of cancer [23, 32–34]. In the current study, we demonstrated a novel mechanism of action of DHA based on its suppression of cell viability of PDAC cells both *in vitro* and *in vivo*. The results showed that DHA effectively inhibited cell viability of human PDAC cells (Fig 1A and 1B) and significantly induced cell cycle arrest at G1 phase by reducing the expression of cyclin E, cyclin A and cyclin B proteins (Fig 1C and 1D). Moreover, the study also displayed that the chemotherapeutic effects of gemcitabine were drastically enhanced when used in combination with DHA so as to inhibit cell viability of PDAC cells (Fig 1E). In this study as DHA effectively inhibited cell viability of human PDAC cells, an interplay of various mechanisms came into action. Firstly, by an increment of GSSG/GSH ratio in PDAC cells (Fig 2A). Secondly, DHA has significantly induced cell apoptosis in PDAC cells (Fig 2B) and thirdly, supplementation of either GSH or NAC effectively reduced DHA-mediated cell death (Fig 2C).

It was seen that DHA has effectively increased the cellular level of cysteine in HPAF-II cells (Fig 3A) and also showed a rise in cellular GSSG/GSH level. As NADPH is required for the conversion of GSSG to GSH, hence treatment with DHA would increase cellular NADP/NADPH ratio causing by depletion of NADPH (Fig 3B). This evidence supports our hypothesis that DHA may inhibit cell viability of PDAC cells by depleting cellular NADPH and increasing GSSG/GSH ratio in PDAC cells.

A previous study has demonstrated that cellular xCT antiporter protein is involved in the transportation of cystine/glutamate. This correlates with our findings where we studied the effects of DHA on the expression of xCT antiporter protein and the results showed that it significantly induced the expression of xCT antiporter and CTH proteins (Fig 3C). Interestingly, DHA slightly suppressed the expression of CBS protein in human PDAC HPAF-II cells (Fig 3C). It’s probable that DHA enhanced the ratio of NADP/NADPH and GSSG/GSH, which led to a feedback control on the increased expression of xCT and CTH proteins in human PDAC HPAF-II cells.

It is well documented that treatment with STAT3 inhibitors induces the expression of xCT protein (Fig 3D). As discussed before, DHA modulates the expression of xCT protein which is associated with a reduced phosphorylation level of STAT3 protein (Fig 3C). Also, HPAF-II cells are featured with TP53 mutation with serine substitution at codon 151 [26]. It was demonstrated in a study that TP53P151S could lead to loss of p53 transcriptional activity and mediate cancer cell proliferation [35]. Similar findings in our study have suggested that DHA induces the expression of xCT protein mainly by blocking STAT3 activation. It was also noticed that pre-treatment with xCT inhibitors including, erastin or SSZ will further inhibit cell viability in DHA (at a dosage of 150 μM)-treated HPAF-II cells (Fig 3E).

This study clearly demonstrated that DHA induces cell cycle arrest at G1 phase, in association with decreased cyclin E, cyclin A and cyclin B levels (Fig 1). It significantly inhibited nucleotide synthesis by reducing cellular levels of UMP, UDP, UTP and CTP (Fig 4A) and also showed decreased cellular ATP level in HPAF-II cells (Fig 4B). Hence, all these findings display a novel anti-cancer mechanism of DHA and suggest that it functions as an effective inhibitor of nucleotide synthesis and bioenergy production.

In order to verify the *in vitro* findings of the study, we examined *in vivo*, whether consumption of FO affects tumor growth and nucleotide synthesis in a mouse xenograft model. The outcome in these experimental mice showed that FO consumption has effectively inhibited tumor growth (Fig 5A and 5B), significantly reduced cellular levels of UTP and CTP (Fig 5C) and has also suppressed cellular ATP levels in tumor tissues (Fig 5D). Our study demonstrated similar results, showed that consumption of FO increases GSSG/GSH ratio in tumor tissues (Fig 5E) and effectively inhibits the expression of CBS protein as compared to the non-FO tumor group (control group) (Fig 5F). Also, it is evident that FO consumption has significantly induced the expression of xCT antiporter and CTH proteins in tumor tissues (Fig 5F).

**Fig 6:**
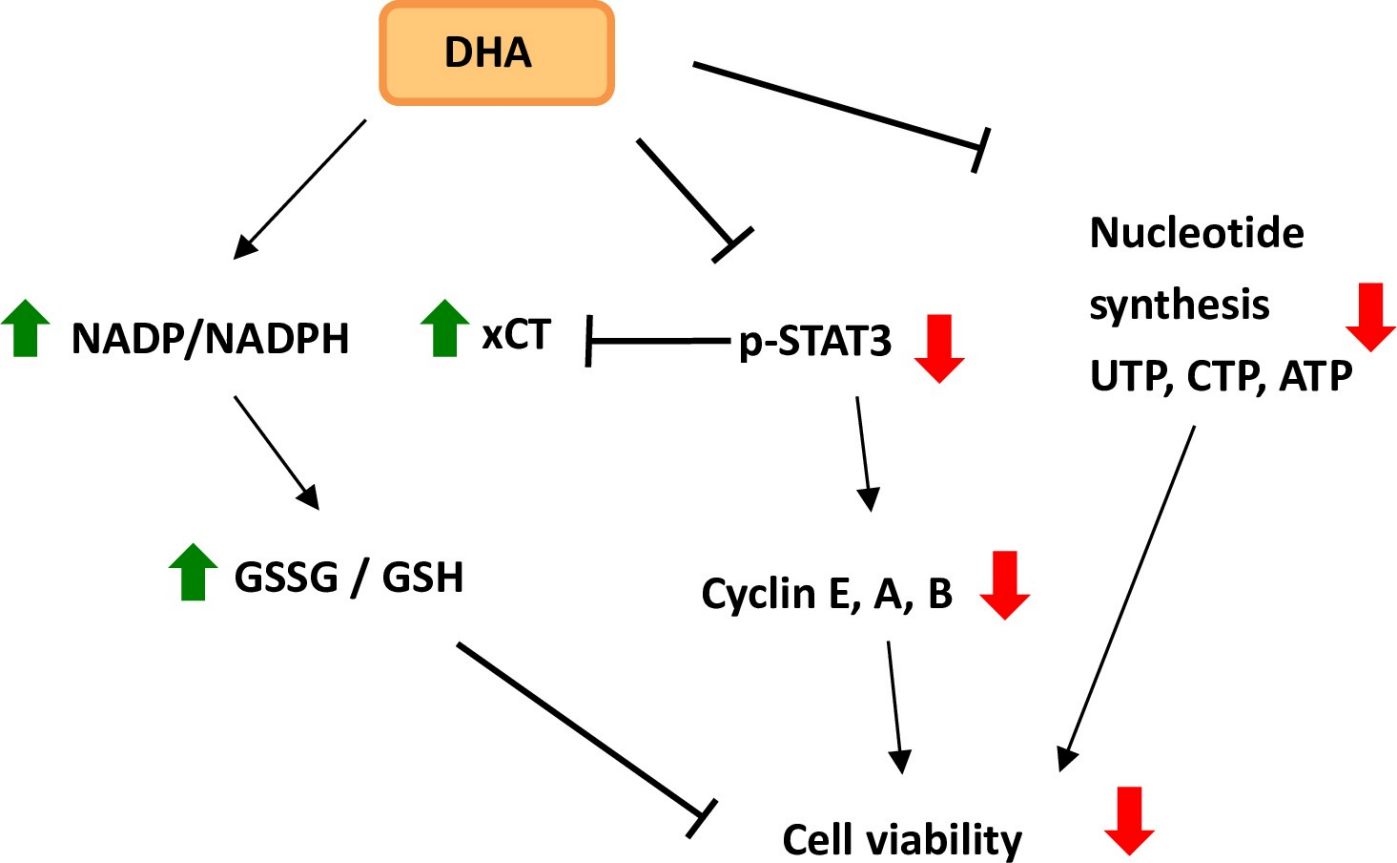
Proposed mechanisms of DHA-mediated modulation of GSSG/GSH and NADP/NADPH ratios and suppression of nucleotide synthesis in human PDAC cells. Red arrows indicate decreased level. Green arrows indicate increased level.→: induction; −│: suppression.

The proposed mechanism in our study suggests that DHA upregulates the NADP/NADPH and GSSG/GSH ratios (Fig 6). DHA inhibited cellular levels of phosphorylated STAT3, cyclin E, cyclin A and cyclin B proteins in human PDAC HPAF-II cells. It also inhibited cell viability by suppressing nucleotide synthesis in HPAF-II cells, both *in vitro* and *in vivo*. In conclusion, these results demonstrate a novel mechanism by which DHA caused suppression of cancer cell survival especially in *Kras* and *TP53* double mutant PDAC cells.

## Supporting information

S1 File (DOCX)
