# Supplementary material for: Docosahexaenoic acid inhibits the proliferation of Kras/TP53 double mutant pancreatic ductal adenocarcinoma cells through modulation of glutathione level and suppression of nucleotide synthesis
Source: PLoS One. 2020 Nov 2;15(11):e0241186. doi: 10.1371/journal.pone.0241186 (PMC7605869; doi:10.1371/journal.pone.0241186)

**S1 Fig. 1D.**

Cyclin D1


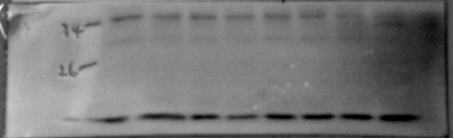


Cyclin E


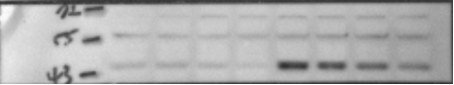


Cyclin A


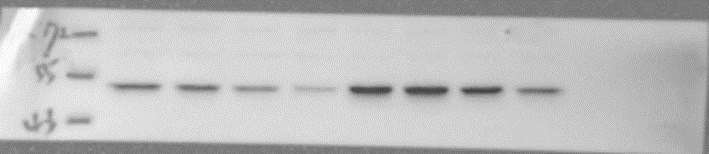


Cyclin B


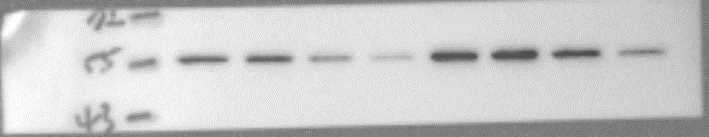


Lamin A


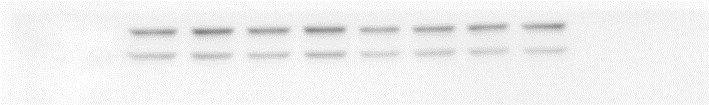


**Fig. 1E**

**
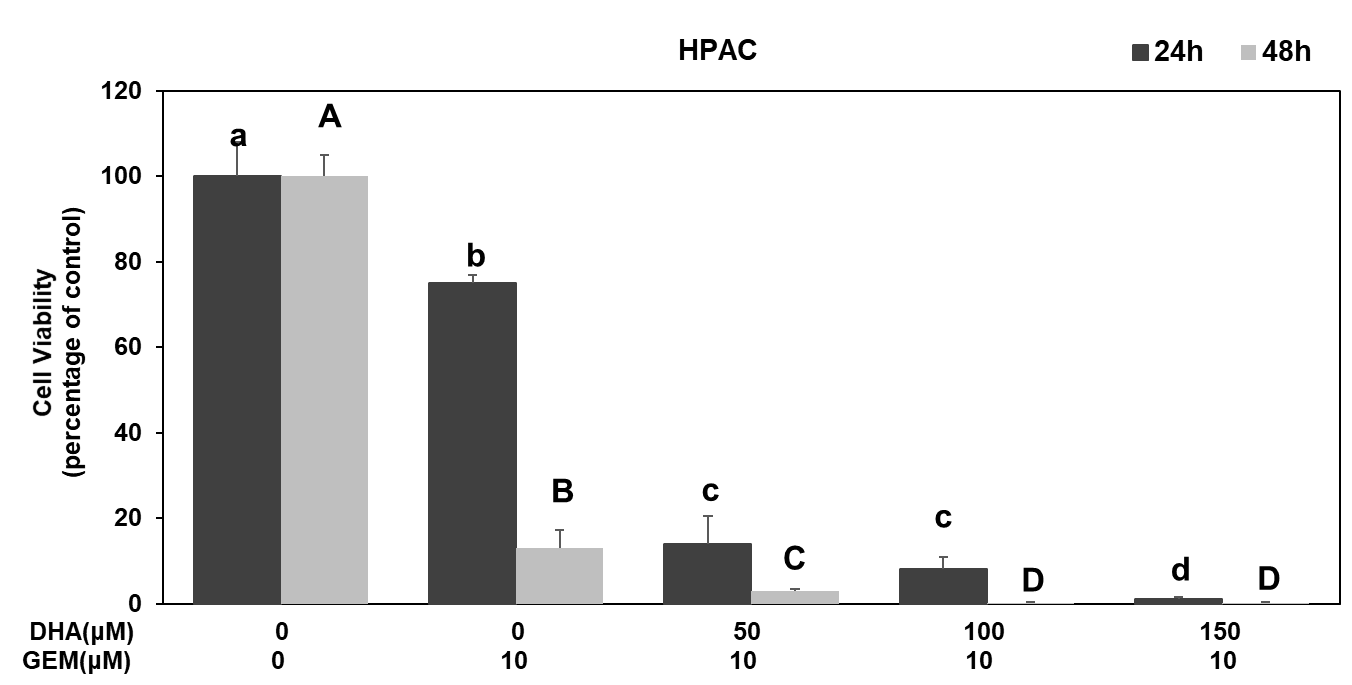
**

Different lower-case letter represents a statistically significant difference within different subgroups at 24 h, at *P* <0.05. Different upper-case letter represents a statistically significant difference within different subgroups at 48 h, at *P* <0.05.

**S2 Fig. 3C.**

p-EGFR


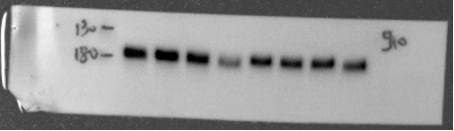


p-c-Met


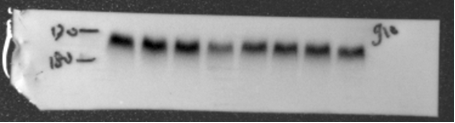


p-STAT3


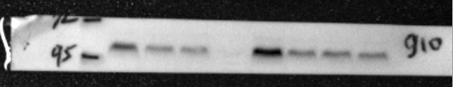


t-STAT3


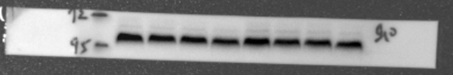


xCT


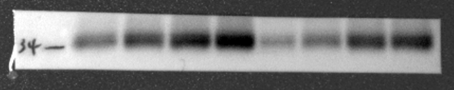


CBS


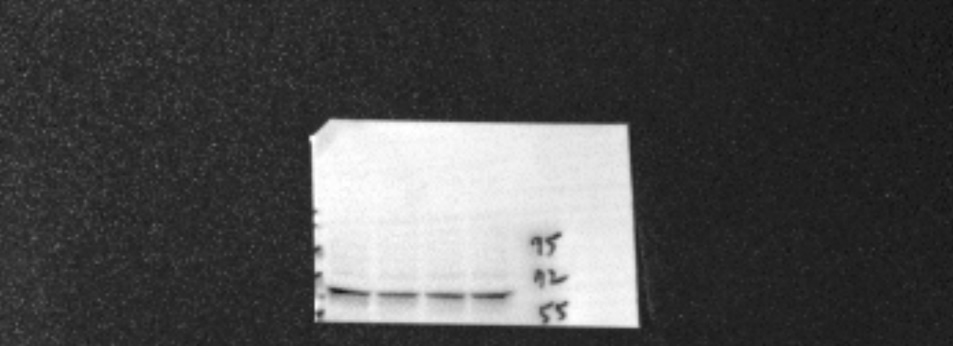


CTH


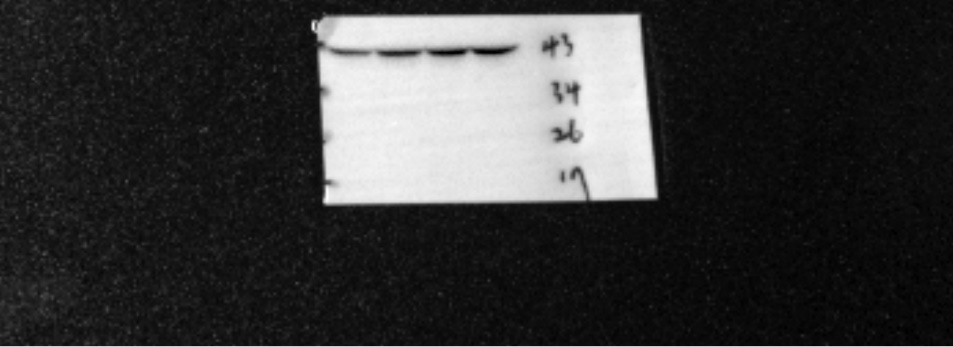


GSS


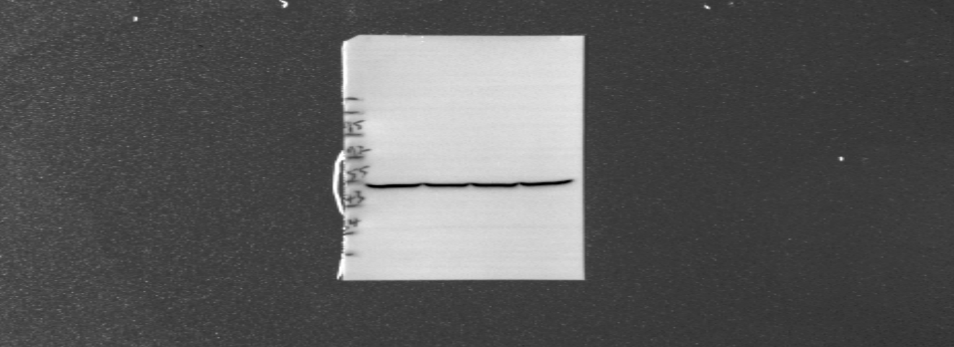


Actin


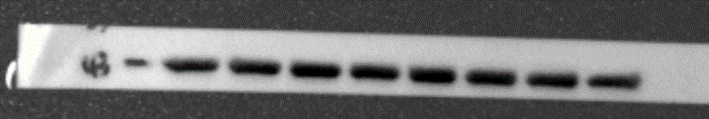


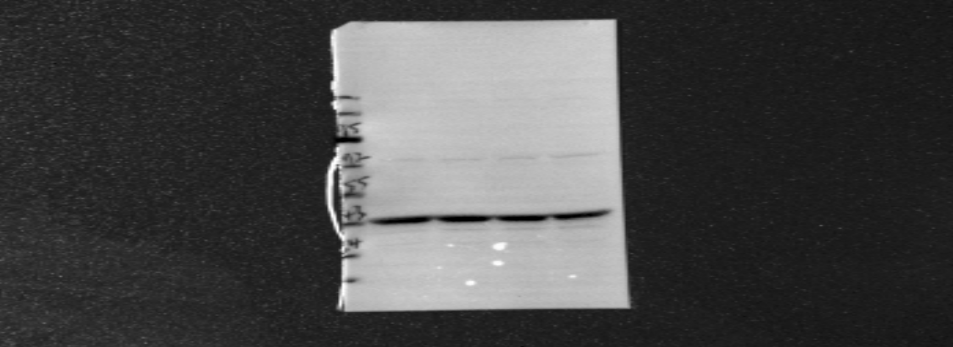


**S2 Fig. 3D.**

p-STAT3


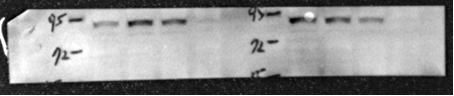


t-STAT3


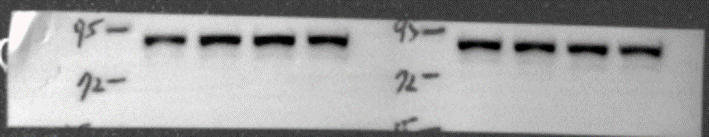


xCT


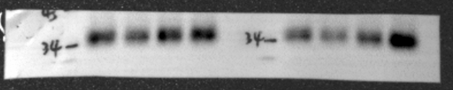


actin


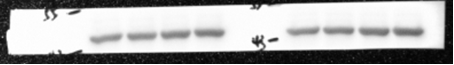


**S3 Fig. 5F.**

xCT


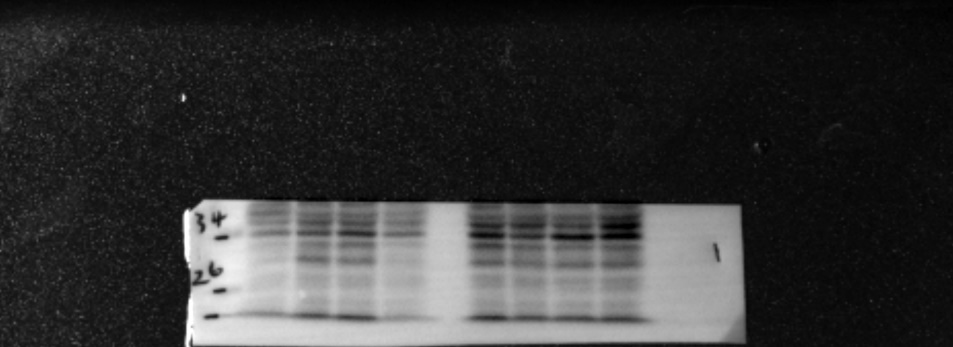


CBS


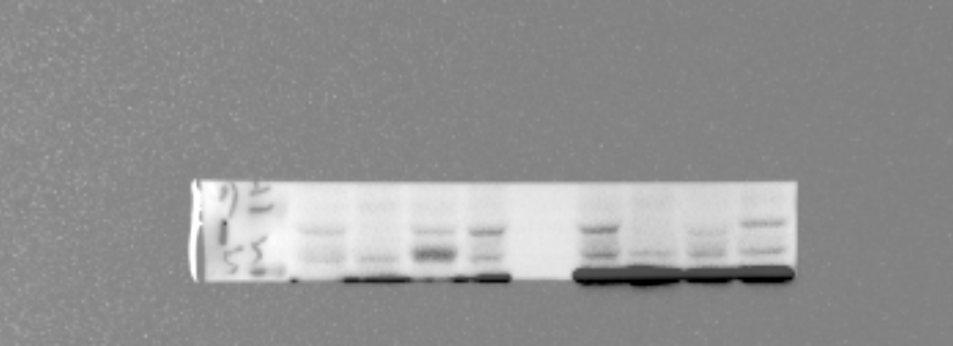


CTH


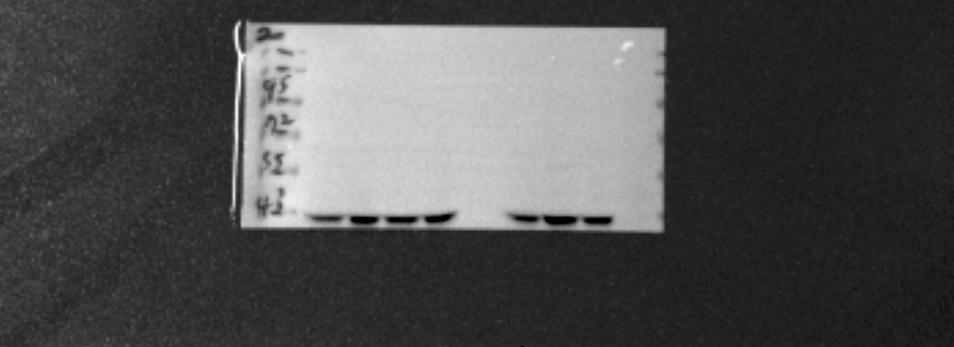


GSS


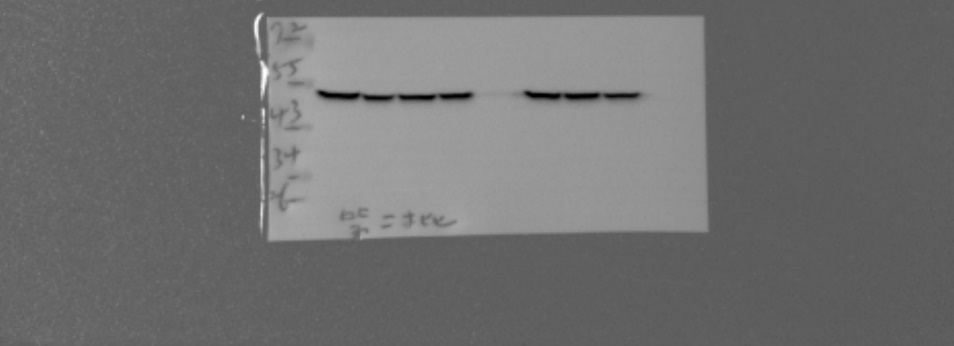


actin


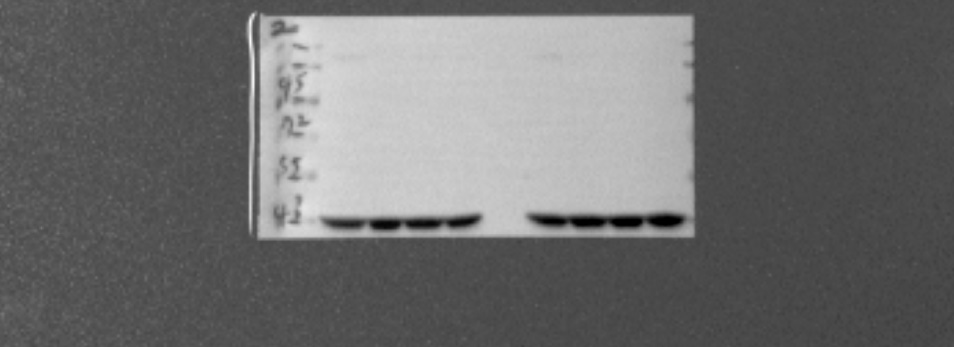

Supplement: S1 File — (DOCX) [file pone.0241186.s001.docx]
